# Shift of N-MYC Oncogene Expression in AML Patients Carrying the FLT3-ITD Mutation

**DOI:** 10.3390/pathophysiology30030024

**Published:** 2023-08-01

**Authors:** Konstantin Bogdanov, Ekaterina Kudryavtseva, Yulia Fomicheva, Irina Churkina, Elza Lomaia, Larisa Girshova, Yuri Osipov, Andrey Zaritskey

**Affiliations:** Almazov National Medical Research Centre, 2 Akkuratova Str., Saint Petersburg 197341, Russia; katrin182002@mail.ru (E.K.); yulya.fomicheva.2015@mail.ru (Y.F.); ichur@mail.ru (I.C.); lomelza@gmail.com (E.L.); lgirshova@gmail.com (L.G.); yosipov88@yandex.ru (Y.O.); zaritskey@gmail.com (A.Z.)

**Keywords:** acute myeloid leukemia, *FLT3-ITD* allelic load, *N-MYC* oncogene expression

## Abstract

Mutations in the *FLT3* gene not only lead to abnormalities in its structure and function, but also affect the expression of other genes involved in leukemogenesis. This study evaluated the expression of genes that are more characteristic of neuroblastoma but less studied in leukemia. *N-MYC* oncogene expression was found to be more than 3-fold higher in primary AML patients carrying the *FLT3-ITD* mutation compared to carriers of other mutations as well as patients with normal karyotype (*p* = 0.03946). In contrast to the expression of several genes (*C-MYC*, *SPT16*, *AURKA*, *AURKB*) directly correlated to the allelic load of *FLT3-ITD*, the expression of the *N-MYC* oncogene is extremely weakly related or independent of it (*p* = 0.0405). Monitoring of *N-MYC* expression in some patients with high *FLT3-ITD* allelic load receiving therapy showed that a decrease in *FLT3-ITD* allelic load is not always accompanied by a decrease in *N-MYC* expression. On the contrary, *N-MYC* expression may remain elevated during the first three months after therapy, which is additional evidence of the emergence of resistance to therapy and progression of AML.

## 1. Introduction

To date, target oncogene mutations arising from chromosomal abnormalities and associated with the course of malignant blood disease, as well as its outcome, have been well-studied. The detection of oncogene defects due to chromosomal abnormalities in acute myeloid leukemia (AML) allows us to divide these disorders into three main groups. The first group of chromosomal aberrations that do not result in the loss of genetic material includes reciprocal chromosomal translocations and inversions, which cause formation of chimeric (fusion) oncogenes. Such changes cause abnormalities in the function of genes encoding transcription factors involved in hematopoiesis. Carrying these gene mutations, taking into account modern treatment regimens, is usually associated with a favorable prognosis [1]. The second group of chromosomal aberrations includes deletions leading to irregular losses of genetic material. Mutational changes in these genes are associated with an unfavorable prognosis, and the pathogenesis of the disease is often caused by dysfunction of anti-oncogenes [2]. Finally, the third group includes cases in which no visible karyotype abnormalities are detected (normal karyotype). As a rule, patients with normal karyotype constitute an intermediate prognostic group. However, this group can be subdivided into different prognostic subgroups that include rare mutations which respond differently to treatment. In particular, detection of the *D816V* mutation in exon 17 of the *C-KIT* gene in 1% of AML patients with normal karyotype correlates with a poor prognosis [3]. In addition, rearrangements of the *MLL* gene are known to occur in 6% of patients with normal karyotype, which is also characterized by an unfavorable course of the disease [4]. Finally, mutations occurring in the FMS-like tyrosine kinase gene *FLT3* (FMS-like tyrosine kinase) are more frequently found in patients with normal and less frequently abnormal karyotypes; for example, in combination with low-risk (*AML1-ETO*, *CBFβ-MYH11/A*, *NPM1/A*) or high-risk mutations (*DEK-NUP214*, *NUP98-NSD1*) [5,6,7,8,9]. Moreover, detection of rare mutations of oncogenes *DEK-NUP214*, *NUP98-NSD1* (<1%) correlates with detection of *FLT3* gene mutations in 90% of cases.

Among mutational changes in the *FLT3* gene, the most common are internal tandem duplications (ITD) detected in the juxtamembrane domain of the *FLT3* gene, and they account for 25–30% of all AML cases, which often correlates with resistance to treatment and an unfavorable prognosis of the disease [7]. Point mutations in the tyrosine kinase domain (TKD) of the *FLT3* gene are less common and account for up to 3–10% of all cases [10]. Their prognostic significance remains unclear. They can be detected both as primary, in the onset of the disease, and secondary, during treatment, which is an indicator of patient resistance to treatment and progression of malignant blood disease [11,12]. It is known that the detection of *FLT3-ITD* mutation in hematopoietic stem cells is associated with inhibition of terminal myeloid differentiation, the appearance of immature cells in the peripheral blood, characterized by the inability to perform their intended functions [13,14]. Apparently, the occurrence of tandem duplications, including *FLT3-ITD*, is associated with the formation of hairpins on the lagging DNA strand caused by replication errors, which leads, in some cases, to the appearance of palindromes [15]. During carcinogenesis, tandem-duplicated genes in the synthesized DNA chain are preserved due to disturbances in the nucleotide mismatch repair system. It is known that, in leukemia, mutations in the *FLT3* gene encoding the receptor protein lead to spontaneous dimerization of the receptor and formation of the enzyme active center, the basis of which is the activation loop (A-loop). Due to the resulting mutations in the *FLT3* gene, constitutive activation of the FLT3 protein occurs and triggers a cascade of reactions that is longer in duration than normal. This contributes to changes in the expression of genes that control the cell cycle and cause an increase in proliferation, block differentiation, and inhibit apoptosis [16]. In particular, *FLT3-ITD* was previously found to activate STAT5, which translocates into the nucleus to bind DNA and express *STAT5*-responsive genes, including *C-MYC* [17]. Recently, the results of determining gene expression, including fusion oncogenes, in 33 adult AML patients using microarray RT-qPCR were presented [18]. In addition to detecting overexpression of several genes, these researchers observed overexpression of the *C-MYC* oncogene in *AML1-ETO* mutation carriers. Previously, another group of scientists found increased expression of the *N-MYC* oncogene in hematopoietic cells of children with AML who were carriers of *AML1-ETO* or *CBFβ-MYH11* mutations [19]. However, in both cases, the potential mechanism of *N-MYC* or *C-MYC* involvement in leukemogenesis is unknown. Nevertheless, by analogy with neuroblastoma and other tumors, it can be assumed that some mitotic serine/threonine kinases (*AURKA*, *AURKB*) and transcription factors (*SPT16*) may be involved to maintain increased expression, particularly of the *N-MYC* oncogene in leukemia. Moreover, these biomarkers control the pericentromeric localization of cohesin, which is involved in DNA replication and ensures sister chromatid cohesion until segregation is imminent at anaphase in mitosis. It should be noted that cohesin subunits knockdown impaired proliferation in neuroblastoma cells expressing *N-MYC* but had no inhibitory effect in control cells [20]. Recently, it became known that cohesin dysfunction is often associated with the detection of *FLT3* gene mutations in AML [21].

At present, the occurrence of the *FLT3-ITD* internal tandem duplication tends to be regarded as a late event in the pathogenesis of AML, while an early event preceding or concomitant with the occurrence of *FLT3* gene mutations remains unknown [22]. The main objective of this work was to quantify the expression of some genes (*C-MYC*, *N-MYC*, *SPT16*, *AURKA*, *AURKB*) that are more characteristic biomarkers for neuroblastoma with poor prognosis, but less studied in AML patients, including carriers of *FLT3-ITD* mutation with high allelic load. Initially, we determined the expression of the *C-MYC* (*WT1*) oncogene in primary patients with AML (*n* = 44) belonging to two different patient groups: *FLT3-ITD*-negative (*n* = 30) and *FLT3-ITD*-positive (*n* = 14). We found a slight excess of the mean level of *C-MYC* (*WT1*) oncogene expression in patients from the *FLT3-ITD*-positive group compared to the *FLT3-ITD*-negative group (*C-MYC*, *p* = 0.005011; *WT1*, *p* = 0.008042). Then, we decided to additionally determine the expression of the *N-MYC* oncogene and the nearest circle of regulatory genes (*SPT16*, *AURKA*, *AURKB*) whose expression is often elevated in neuroblastoma and remains unstudied in adult patients with AML, including *FLT3-ITD* mutation carriers. According to the present work, there was a significant, more than 3-fold higher, level of *N-MYC* oncogene expression in *FLT3-ITD* mutation carriers compared to *FLT3-ITD*-negative group of patients. In addition, it was required to find out whether at least one of the studied biomarkers could be used to assess minimal residual disease (MRD) in some patients (*n* = 4), *FLT3-ITD* mutation carriers, during the first three months of receiving therapy.

## 2. Materials and Methods

### 2.1. Ethics and Experimental Design

The study was carried out in compliance with the principles of the Helsinki Declaration of the World Medical Association with the consent of the Ethics Committee of the Federal State Budgetary Institution “Almazov National Medical Research Centre” of the Ministry of Health of the Russian Federation (conclusion of 31 October 2019). According to the research, all patients were divided into two groups based on the results of *FLT3-ITD* mutation detection.

#### 2.1.1. Inclusion Criteria

The study was conducted in primary patients (*n* = 44) with acute myeloid leukemia (AML) who were diagnosed in the Almazov National Medical Research Centre. The average age of the patients was 39 (18–72) years. Information on the comorbidities and sex of the study patients is presented in Table S1. All bone marrow (BM) samples were taken at the onset of the disease. Some of them (*n* = 4) were taken for monitoring, if treatment permitted.

#### 2.1.2. Exclusion Criteria

Patients with acute promyelocytic leukemia (APL) were excluded from the study because there were no primary patients among them.

### 2.2. RNA Extraction and cDNA Synthesis

RNA was isolated from 1 mL of total BM cells using the QIAamp RNA Blood Mini Kit (Qiagen, Hilden, Germany). Lysis solution was added to the cell suspension and further processing was performed according to the protocol. RNA was eluted in 30 µL of RNase-free buffer. The reverse transcription (RT) reaction was carried out according to the procedure for cDNA synthesis using the RevertAid H Minus First Strand cDNA Synthesis Kit (Fermentas, Vilnius, Lithuania).

### 2.3. DNA Extraction

gDNA was isolated from 0.2 mL of total BM cells using the QIAamp DNA Mini Kit (Qiagen) according to the manufacturer’s protocol. DNA was eluted in 50 μL of buffer AE (10 mM Tris·Cl, 0.5 mM EDTA, pH 9.0).

### 2.4. Screening of Target Oncogene Mutations, including FLT3 Gene Mutations

To detect various oncogene mutations (*AML1-ETO*, *CBFβ-MYH11*, *PML-RARα*, *BCR-ABL*, *DEK-NUP214*, *NUP98-NSD1*, *MLL-AF4*), cDNA was amplified using microarray real-time PCR after reverse transcription (RT) [23]. Each sample for PCR contained 1× PCR buffer, 2.5 mM of each dNTP, 10 pM forward and reverse primers, 6 pM FAM-labeled probe at the 5′-end, 10 ng of cDNA, and 5 units of Taq polymerase (Evrogen, Moscow, Russia). Commercially synthesized oligonucleotides were the same as those published previously [24,25,26,27]. Thermocycling steps included the initial denaturation at 95 °C for 120 s followed 50 cycles: 95 °C for 3 s and 60 °C for 30 s were performed on an AriaDNA amplifier (Lumex, St. Petersburg, Russia).

To detect target mutations in the *NPM1*, *C-KIT*, and *FLT3* genes, amplification of gDNA was performed on a DNA Engine Dyad Peltier Thermal Cycler (Bio-Rad, Hercules, CA, USA). Each PCR sample contained 1× PCR buffer, 2.5 mM of each dNTP, 10 pM forward and reverse primers, 100 ng of gDNA, and 5 units of Encyclo Taq polymerase (Evrogen, Moscow, Russia). Commercially synthesized oligonucleotides and thermal cycling conditions were the same as for PCR followed by direct Sanger sequencing. Mutations in exons 14, 15, and 20 of the *FLT3* gene were determined using PCR and agarose gel electrophoresis. To confirm the mutations detected, direct Sanger sequencing was performed using an ABI PRISM 3110 genetic analyzer (Applied Biosystems, Waltham, MA, USA) as described previously [28]. The mutations localized in exon 12 of the *NPM1* gene and exon 17 of the *C-KIT* gene were analyzed by direct Sanger sequencing as presented before [29,30].

### 2.5. Determination of the Allelic Load of FLT3 Gene Mutations

To determine the allelic load of the *FLT3-ITD* (*FLT3-TKD*) mutation, a DNA sample (2–4 ng) was amplified using PCR. It should be noted that the PCR conditions were the same as described above, except that each sample contained 1 pM of forward and reverse primers (the forward primer was labeled 6-FAM at the 5′-end). Commercially synthesized oligonucleotides and thermal cycling conditions were the same as those specified for PCR followed by Sanger direct sequencing [28]. To detect the allelic load of the *FLT3-TKD D835* mutation, PCR products were digested with EcoRV restriction enzyme before capillary electrophoresis. After fragment analysis was performed, the peak areas for wild type and mutation were estimated using software for the Nanofor-05 sequencer (Helicon, Moscow, Russia).

### 2.6. Quantitative Gene Expression Analysis

To quantify mRNA transcripts of the tested genes (*WT1*, *SPT16*, *AURKA*, *AURKB*, *N-MYC*, *C-MYC*), the target oncogenes (*CBFβ-MYH11/A*, *AML1-ETO*, *NPM1/A*), and the *GAPDH* reference gene, cDNA samples were amplified by RT-qPCR. To determine the gene expression of *WT1*, *CBFβ-MYH11/A*, *AML1-ETO*, and *NPM1/A*, RT-qPCR was performed using the Fusion Quant Kit (Qiagen), the Universal PCR Master Mix (Applied Biosystems), and commercially synthesized oligonucleotides, whose nucleotide sequences, together with the RT-qPCR protocol, have been published previously [24,25,31,32]. Expression of *N-MYC*, *C-MYC*, *SPT16*, *AURKA*, and *AURKB* genes was assessed by RT-qPCR using the Universal PCR Master Mix Kit (Applied Biosystems) and commercially synthesized oligonucleotides that were selected using the NCBI system (Table S2). Each PCR sample contained 1× Universal PCR master mix, 10 pM forward and reverse primers, 6 pM FAM-labeled probe at the 5′-end, and 100 ng of cDNA. Thermocycling steps included holding at 50 °C for 2 min, and holding at 95 °C for 10 min, followed by 50 cycles: 95 °C for 15 s and 60 °C for 1 min. The PCR results were analyzed using the RotorGene 6000 thermal cycler software (Corbett Research, Sydney, Australia) after generating calibration curves with standard dilutions of plasmid DNA (10^1^–10^6^). The threshold levels of quantitative gene expression of *WT1*, *AURKA*, *AURKB*, *SPT16*, *N-MYC*, and *C-MYC* in BM cells of healthy donors (*n* = 10) did not exceed 2.5 × 10^2^, 9.2 × 10^2^, 1.0 × 10^3^, 8.6 × 10^4^, 2.3 × 10^5^, and 4.5 × 10^5^ copies/*GAPDH*. An excess of these values was considered as an increase in expression.

### 2.7. Cytogenetic Study

Preparation of chromosome spreads and subsequent chromosome differential staining were performed according to the assay described previously [33]. The karyotype pathology was interpreted by analysis of 20 mitoses by standard karyotyping and/or 200 interphase nuclei after fluorescence in situ hybridization (FISH).

### 2.8. Statistical Analysis

Comparative analysis of the average expression levels of the genes under study in two different groups of patients was performed using a one-way ANOVA with Bonferroni’s post-test for multiple analysis. The relationship between the relative expression of the genes under study and the allelic load of the *FLT3-ITD* mutation was assessed after plotting the XY correlation plot with the line of best fit and determining the linear regression coefficient. The correlation association between the insertion length (allelic load value) of the *FLT3-ITD* mutation and the achievement of remission (disease outcome) in patients from two different groups was assessed using the χ^2^ test.

## 3. Results

### 3.1. Analysis of Oncogene Mutations in Primary AML Patients

According to the cytogenetic study performed in primary patients with AML (*n* = 44), an abnormal karyotype was detected in 21 patients, while the karyotype of the remaining 23 patients was normal. Molecular genetic screening of target oncogene mutations was also performed. According to the mutations identified, all patients were divided into two groups (Table 1). The *FLT3-ITD*-negative group (*n* = 30) consisted of patients who had no mutations in the *FLT3* gene but one of the other two mutations was detected: *AML1-ETO* [t(8;21)(q22;q22)] (*n* = 13), *CBFβ-MYH11/A* [inv16(p13;q21)] (*n* = 8), and patients with normal karyotype (*n* = 9), in whom no mutations were identified. Of note, that 5 of the 30 patients mentioned above had the *NPM1/A* gene mutation, 3 of whom were *AML1-ETO* carriers and two others were *CBFβ-MYH11/A* carriers. The *FLT3-ITD*-positive group (*n* = 14) consisted of patients with normal karyotype who were carriers of *FLT3* gene mutations: *FLT3-ITD* or *FLT3-ITD/FLT3-TKD*. The *FLT3-TKD* mutation was detected in one patient who was also a carrier of the *FLT3-ITD* mutation. The frequency of the *FLT3-TKD* mutation among the AML patients studied was 2.3%, whereas the frequency of the *FLT3-ITD* mutation did not exceed 31.8%. Of note, 7 of the 14 *FLT3-ITD* carriers had the *NPM1/A* gene mutation (Table 1 and Table S3). In all *FLT3-ITD* mutation carriers, the duplication insertion length was determined as previously described [34]. In total, 8 of the 14 *FLT3-ITD* carriers had an insertion length greater than 39 bp, whereas the remaining 6 patients had an insertion length shorter than 39 bp. The present study showed no significant differences with respect to complete hematological remission (CHR) or mortality in *FLT3-ITD* carriers with different insertion lengths: >39 bp vs. <39 bp (Table 2).

### 3.2. Determination of Allelic Load of FLT3-ITD Gene Mutation

In addition, the allelic load of the *FLT3-ITD* mutation was determined in all patients belonging to the *FLT3-ITD*-positive group. A total of 6 patients had an allelic load below 0.5 (0.15–0.49), whereas the remaining 8 patients had an allelic load above 0.5 (0.64–6.3). At the same time, carriers of the *FLT3-ITD* mutation with a lower (<0.5) allelic load achieved CHR more frequently than carriers with a higher (>0.5) allelic load (Table 2). Of note, in 1 patient who had a low (0.45) allelic load at baseline, a course of induction therapy followed by therapy with the tyrosine kinase inhibitor gilteritinib (120 mg/day) resulted in the achievement of CHR and a complete molecular response. However, the patient subsequently developed pulmonary failure due to SARS-CoV-2 infection, followed by death.

### 3.3. Relative Expression of the Studied Genes in Two Different Groups of AML Patients

**Figure 1:**
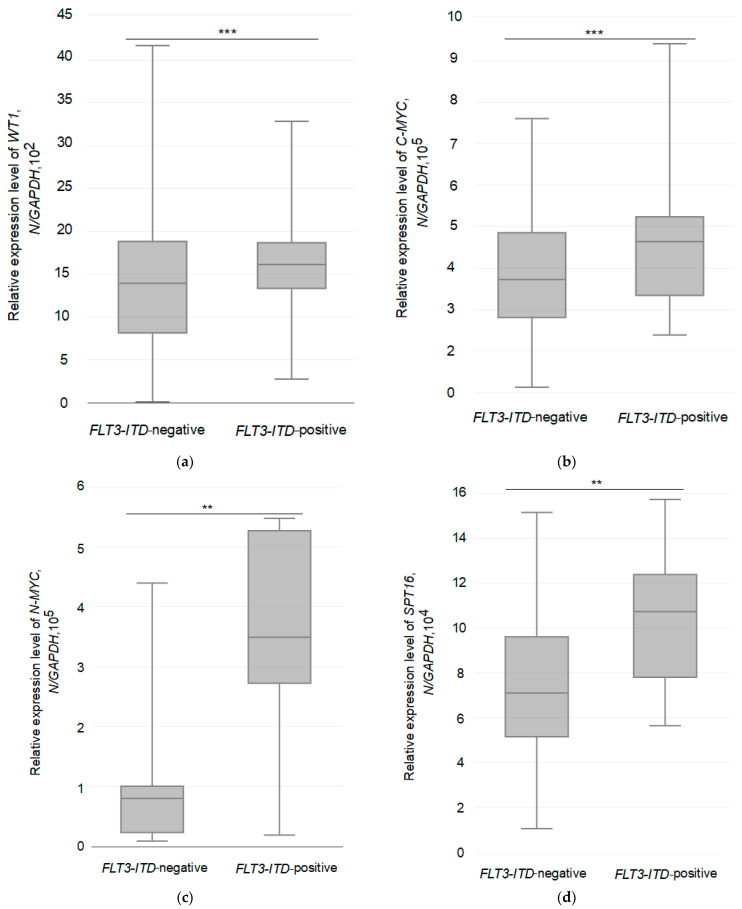

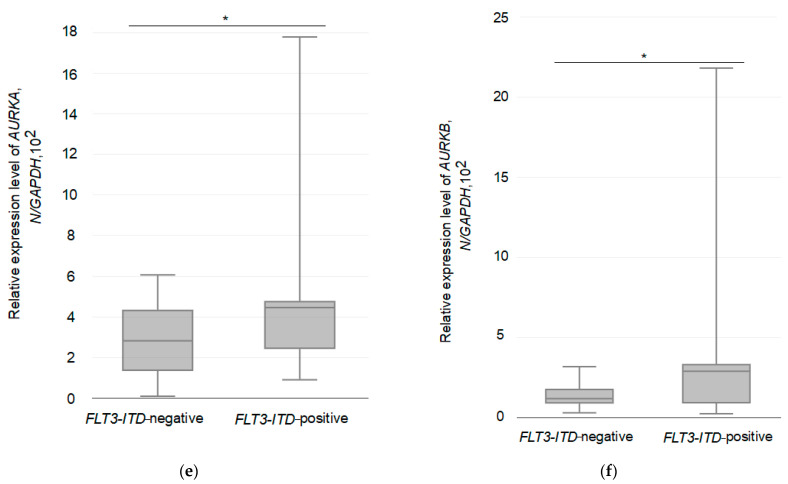
Range of changes in relative expression level of the studied genes in different groups in AML patients. (**a**,**b**) *** *p* < 0.01, (**c**,**d**) ** *p* < 0.05, (**e**,**f**) * *p* > 0.05, the above *p*-values were calculated by one-way ANOVA with Bonferroni’s post-test for multiple analysis, which shows the difference between the patient groups studied: *FLT3-ITD*-negative and *FLT3-ITD*-positive (Table 3).

The expression levels of several genes, including *WT1*, *C-MYC*, *N-MYC*, *SPT16*, *AURKA*, and *AURKB*, were quantitatively analyzed in all primary AML patients. The differences in gene expression data between the groups are shown in Figure 1a–f. According to the results of comparative analysis, the average expression levels of *WT1* and *C-MYC* oncogenes were higher in *FLT3-ITD* mutation carriers: 1.6 × 10^3^/*GAPDH* and 4.6 × 10^5^/*GAPDH* (Table 3). Whereas, in the remaining patients belonging to the *FLT3-ITD*-negative group, the average expression level of *WT1* and *C-MYC* oncogenes was lower and did not exceed the following values: 1.4 × 10^3^/*GAPDH* and 3.8 × 10^5^/*GAPDH*. Moreover, the average level of *N-MYC* oncogene expression was also higher in *FLT3-ITD* mutation carriers (3.4 × 10^5^/*GAPDH*) than in the group of *FLT3-ITD*-negative patients (0.8 × 10^4^/*GAPDH*). However, in contrast to the average expression levels of *WT1* and *C-MYC* oncogenes, the average expression level of the *N-MYC* oncogene was markedly different in the *FLT3-ITD*-positive group compared to the negative group, exceeding it more than 3-fold, as shown in Figure 1a–c. Of note, *N-MYC* oncogene overexpression was detected in 2 *FLT3-ITD*-negative patients carrying *AML1-ETO* and in the majority (11 of 14, 78.5%) of *FLT3-ITD*-positive patients. Elevated *C-MYC* oncogene expression was detected in 13 AML patients, including *FLT3-ITD*-negative (*AML1-ETO*, *n* = 5; *CBFβ-MYH11/A*, *n* = 2; normal karyotype, *n* = 2) and *FLT3-ITD*-positive (*n* = 4) patients. In contrast, increased expression of the *WT1* oncogene was detected in the majority of AML patients (39 of 44), regardless of the group to which they belonged.

As for the average expression levels of the *SPT16*, *AURKA*, and *AURKB* genes in the two different groups, they were highest in the *FLT3-ITD*-positive (1.0 × 10^5^/*GAPDH*, 4.2 × 10^2^/*GAPDH* and 3.1 × 10^2^/*GAPDH*) group (Table 3). In the *FLT3-ITD*-negative group, the mean expression levels of the *SPT16*, *AURKA*, and *AURKB* genes were lower and did not exceed the following values: 7.7 × 10^4^/*GAPDH*, 2.8 × 10^2^/*GAPDH*, 1.3 × 10^2^/*GAPDH*. The range of changes in the expression of the studied genes in both groups is shown in Figure 1d–f. Increased expression of *SPT16* was detected in *FLT3-ITD*-positive (*n* = 9) and *FLT3-ITD*-negative (*AML1-ETO*, *n* = 5; *CBFβ-MYH11/A*, *n* = 4; normal karyotype, *n* = 2) patients. *AURKA/B* overexpression was detected in 1 patient carrying the *FLT3-ITD* mutation. Thus, despite a slight (*WT1*, *C-MYC*, *AURKA*, *AURKB*) or pronounced (*SPT16*, *N-MYC*) increase in the average expression level of the above mentioned genes in the *FLT3-ITD*-positive group compared to the *FLT3-ITD*-negative group, the difference between them was statistically significant only for four genes: *WT1*, *C-MYC*, *N-MYC*, and *SPT16* (Table 3).

### 3.4. Relationship between the Expression of the Tested Genes and the Allelic Load of the FLT3-ITD Mutation

**Figure 2:**
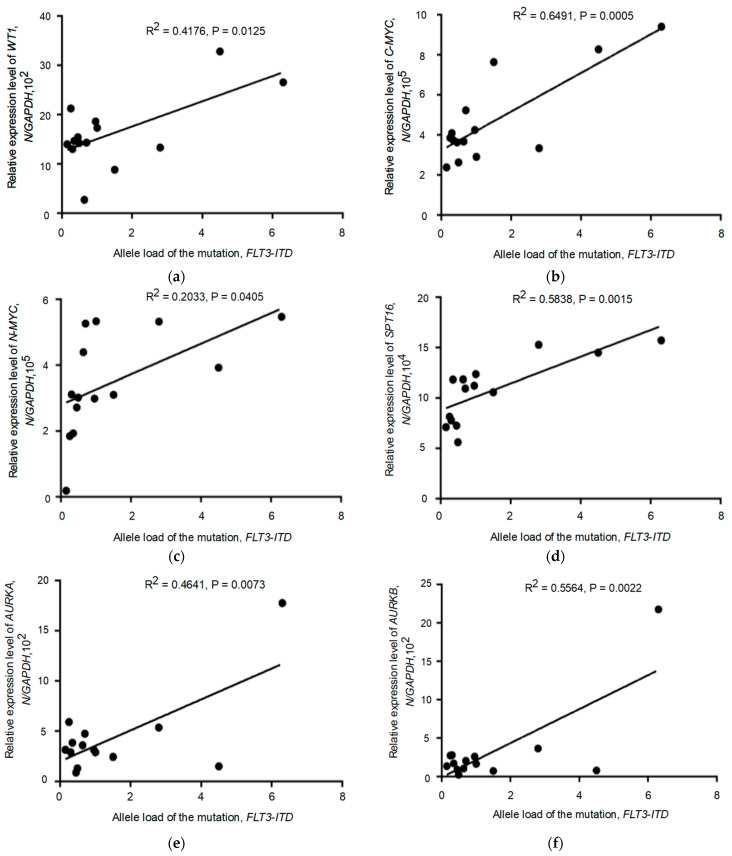
Evaluation of the relationship between the relative expression level of the studied gene and the value of *FLT3-ITD* allelic load in AML patients carrying the *FLT3-ITD* mutation. (**a**–**f**) Relative expression level of *WT1*, *C-MYC*, *N-MYC*, *SPT16*, *AURKA*, *AURKB* genes in *FLT3-ITD* mutation carriers.

Correlation analysis was performed to determine the relationship between the relative expression level of the studied genes (*WT1*, *C-MYC*, *N-MYC*, *SPT16*, *AURKA*, *AURKB*) and the allelic load value of the *FLT3-ITD* mutation in the *FLT3-ITD*-positive group (Table S3). The results are shown in Figure 2a–f.

**Figure 3:**
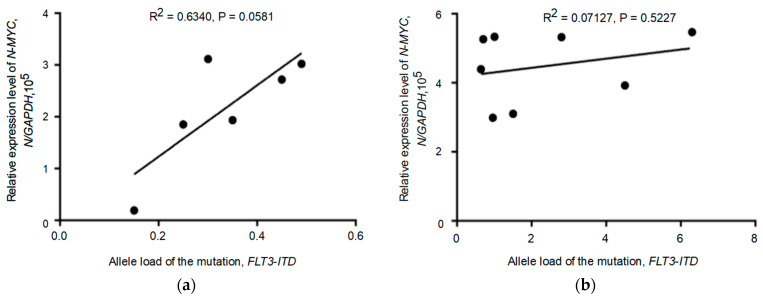
Evaluation of the relationship between the relative level of *N-MYC* oncogene expression and *FLT3-ITD* allelic load in patient subgroups with different allelic load thresholds (>0.5 vs. <0.5). (**a**,**b**) Diving patients carrying the *FLT3-ITD* mutation into two subgroups based on the detection of a high (>0.5) or low (<0.5) allelic load showed no significant association between *N-MYC* expression and *FLT3-ITD* allelic load in the patient subgroups studied.

The expression levels of five genes (*WT1*, *C-MYC*, *SPT16*, *AURKA*, *AURKB*) were shown to correlate with increased *FLT3-ITD* allelic load (*p* = 0.0125, *p* = 0.0005, *p* = 0.0015, *p* = 0.0073, *p* = 0.0022). As for the *N-MYC* oncogene, correlation analysis showed a weak or absent association between *N-MYC* gene expression and *FLT3-ITD* allelic load in patients carrying the *FLT3-ITD* mutation (*p* = 0.0405). We then analyzed patients in two subgroups with *FLT3-ITD* allelic load thresholds above and below 0.5 (>0.5 vs. <0.5). In this case, we found no significant correlation between *N-MYC* expression and *FLT3-ITD* allelic load in both patient subgroups, as shown in Figure 3a,b.

**Figure 4:**
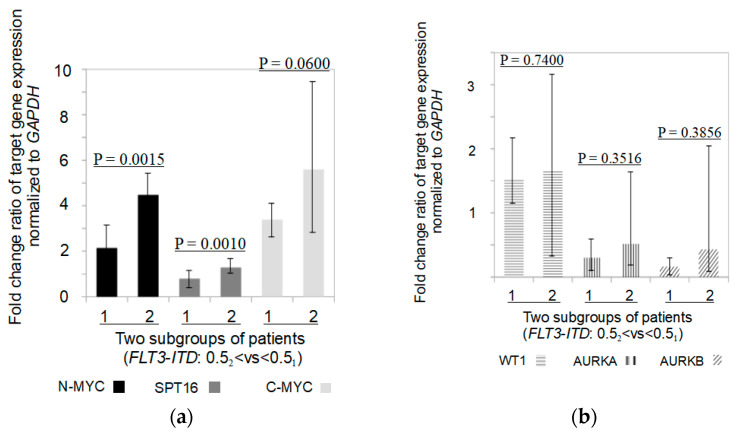
Fold change of gene expression normalized to *GAPDH* in two subgroups of patients with different allelic load of *FLT3-ITD* mutation (>0.5 vs. <0.5). (**a**) Fold change of *N-MYC, C-MYC, SPT16*, (**b**) *WT1, AURKA, AURKB* gene expression in studied subgroups of patients.

We also determined the fold change of gene expression in patients carrying the *FLT3-ITD* mutation depending on allelic load. As a result, we found 1.1, 1.5, 1.6, 1.7, 2.0, and 2.6-fold increases in gene expression levels of *WT1*, *SPT16*, *AURKA*, *C-MYC*, *N-MYC*, and *AURKB* in *FLT3-ITD* mutation carriers with higher (>0.5) allelic load compared with *FLT3-ITD* carriers with lower (<0.5) allelic load, as shown in Figure 4a,b.

### 3.5. Biomarker Monitoring in FLT3-ITD Carriers Receiving Therapy

**Figure 5:**
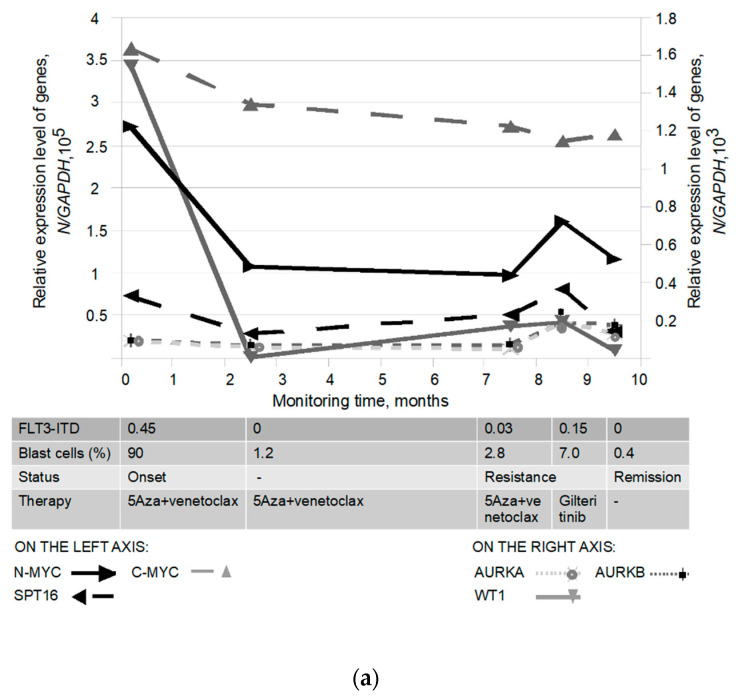

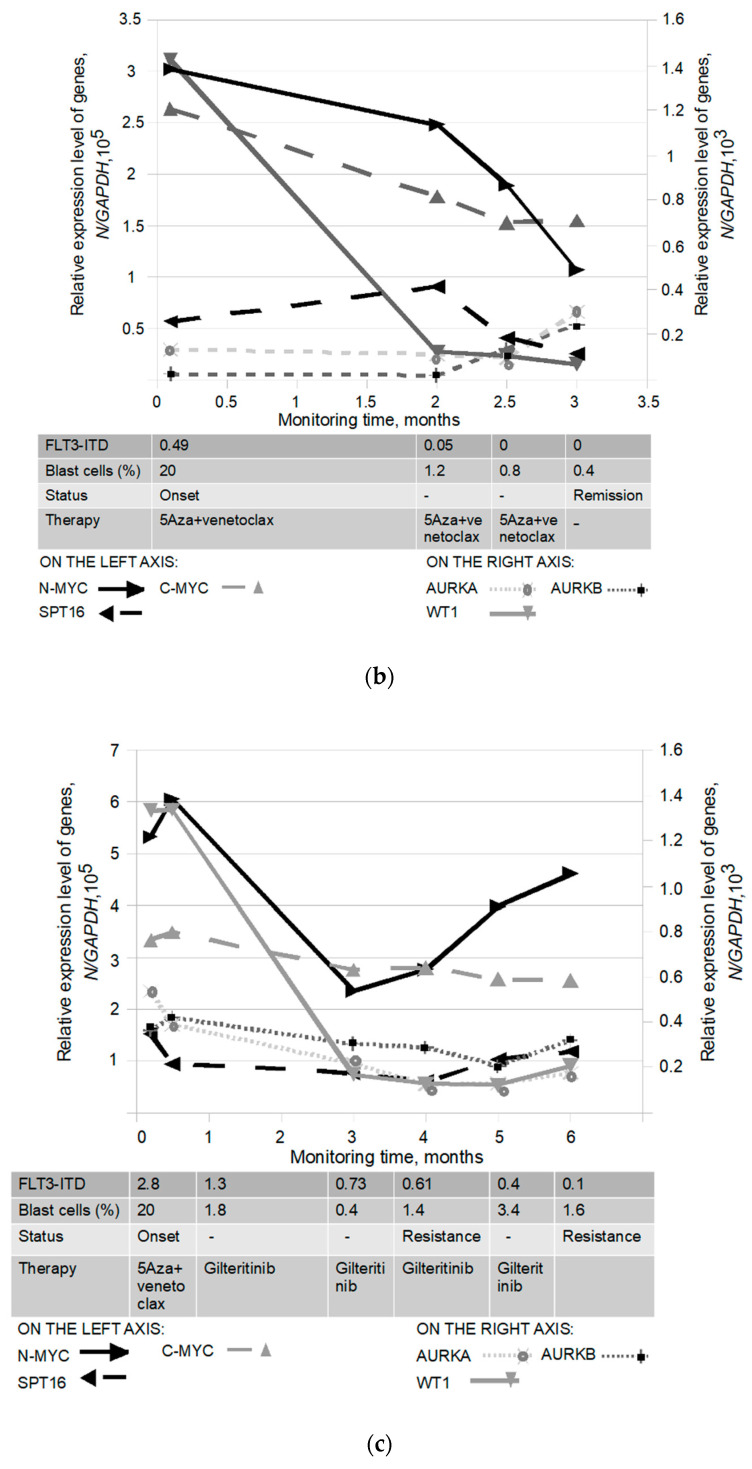

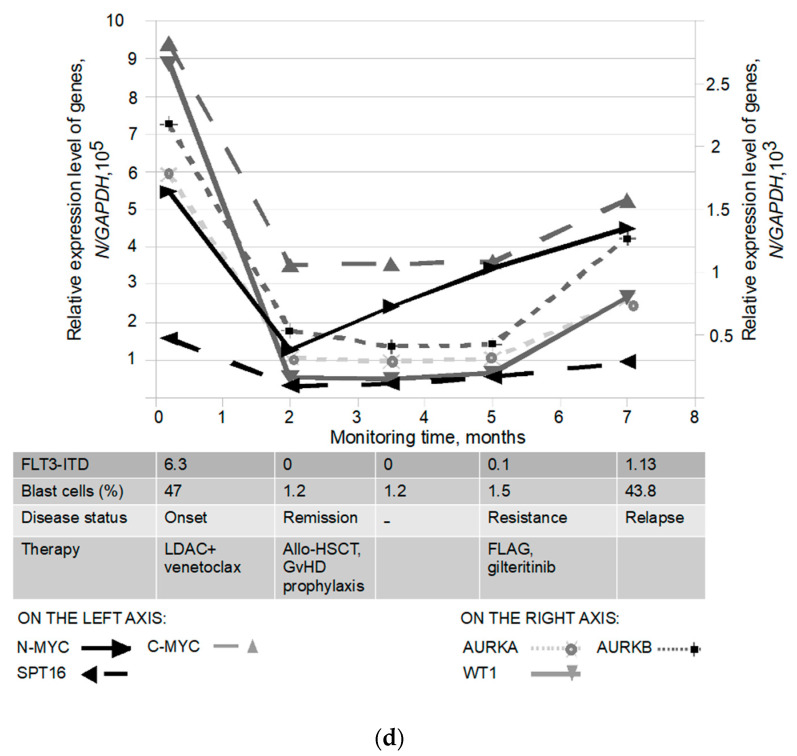
Monitoring of biomarkers in AML patients; carriers of the *FLT3-ITD* mutation after therapy. (**a**,**b**) Quantification of gene expression in some patients with low or (**c**,**d**) high allele load of *FLT3-ITD* mutation.

In addition, we assessed the allelic load of the *FLT-ITD* mutation and the expression levels of the studied genes (*WT1*, *C-MYC*, *N-MYC*, *SPT16*, *AURKA*, *AURKB*) in four patients who received therapy. The results of monitoring in the above patients (A, B, C, D) are presented in Table S4 and Figure 5a–d.

According to Figure 5a,b, two patients (A and B) had a low (0.45 and 0.49) *FLT3-ITD* allelic load in the onset of AML and simultaneously overexpressed *N-MYC* (2.7 and 3.0 × 10^5^/*GAPDH*) and *WT1* (1.5 and 1.4 × 10^3^/*GAPDH*) oncogenes. After therapy, both patients achieved CHR and full molecular response. All investigated biomarkers of both patients returned to normal, including a decrease in *N-MYC* (*WT1*) oncogene expression. However, one of these patients (A), as mentioned above, was infected with SARS-CoV-2 one month after achieving remission, resulting in pulmonary failure and death. As shown in Figure 5c,d, the other two patients (C and D) initially had a high (2.8 and 6.3) *FLT3-ITD* allelic load and *N-MYC* overexpression (5.3 and 5.4 × 10^5^/*GAPDH*). Moreover, both patients had increased expression of the *WT1* (1.3 and 2.6 × 10^3^/*GAPDH*) and *SPT16* (1.52 and 1.57 × 10^5^/*GAPDH*) genes. However, only one of these patients had elevated *AURKA* and *AURKB* gene expression (1.7 and 2.1 × 10^3^/*GAPDH*). The first patient (C) received the tyrosine kinase inhibitor gilteritinib (120 mg/day; hematopoietic stem cell transplantation was not recommended because of cardiac parameters) after induction therapy. The patient achieved hematological remission with partial recovery of peripheral blood (platelet counts) and simultaneous reduction of *FLT3-ITD* allelic load (0.1). During 3 months of therapy, the expression of *WT1*, *C-MYC*, *AURKA*, *AURKB* decreased and did not exceed normal levels, whereas the expression of *N-MYC* (*SPT16*) increased. Four months later, the patient relapsed and died. The second patient (D) received myeloablative conditioning, allogeneic hematopoietic stem cell transplantation (allo-HSCT), and chemotherapy. During 3 months after transplantation, the *FLT3-ITD* allelic load was undetectable or reduced. At the same time, the expression of *WT1*, *C-MYC*, *AURKA*, *AURKB*, and *SPT16* did not exceed normal values. On the contrary, *N-MYC* expression gradually increased. Five months after transplantation, the patient relapsed (blasts: 43.8%; *FLT3-ITD*: 1.13), resulting in a fatal outcome. Of note, *N-MYC* expression was significantly elevated in both patients after therapy, despite decreased *FLT3-ITD* allelic load. At the same time, the development of treatment resistance was observed in both patients.

## 4. Discussion

Initially, target oncogene mutations including *FLT3* gene mutations were identified in the majority of primary AML patients (*n* = 35). Validation showed that the frequency of detected mutations did not differ from the data of other studies [35,36,37]. All patients were divided into two different groups. The first, *FLT3-ITD*-negative group consisted of carriers of other mutations (*AML1-ETO*, *CBFβ-MYH11/A*) and patients without detectable mutations (normal karyotype). The second, *FLT3-ITD*-positive group consisted of carriers of *FLT3* gene mutations. In the *FLT3-ITD*-positive group of patients, we additionally assessed the allelic load and insertion length of *FLT3* gene mutations. This study showed that patients with a lower (<0.5) *FLT3-ITD* allele load achieved CHR significantly more often than patients with a higher (>0.5) *FLT3-ITD* allele load (Table 1). This is consistent with data published previously [38]. However, we found no significant difference between subgroups of patients carrying a shorter or longer insertion (>39 bp vs. <39 bp) of the *FLT3-ITD* mutation with respect to achieving complete remission and the occurrence of fatal outcomes (Table 1). This conclusion is supported by analysis of larger patient populations in subgroups [39].

In addition, the expression levels of *WT1*, *C-MYC*, *N-MYC*, *SPT16*, *AURKA*, and *AURKB* genes were determined in all patients belonging to two different groups. The analysis showed that the average level of *WT1* oncogene expression was higher in *FLT3-ITD* mutation carriers compared to *FLT3-ITD*-negative patients, carriers of other mutations (*AML1-ETO*, *CBFβ-MYH11*), or patients with normal karyotype (*p* = 0.0125). This is consistent with previously published data [40,41]. According to our analysis, overexpression of *WT1* was detected in the majority (39 of 44, 88.6%) of patients studied. Increased expression of *WT1* oncogene detected in the debut of AML is known to be an important prognostic indicator. Monitoring the expression of this biomarker makes it possible to determine MRD in patients receiving therapy, as well as to assess the risk of relapse [42].

According to the present study, there was an increase in the average level of *C-MYC* oncogene expression in both groups among patients with primary AML. However, the difference between the average levels of *C-MYC* expression in these groups was poorly pronounced. *C-MYC* overexpression was detected in 13 AML patients; 4 of whom were carriers of the *FLT3-ITD* mutation and the remaining 9 patients were carriers of other mutations (*AML1-ETO* or *CBFβ-MYH11*) or had normal karyotype. It should be noted that no unfavorable cytogenetic abnormalities that could lead to increased expression of the *C-MYC* oncogene were detected among all the patients studied, as previously shown [43]. One of the causes of *C-MYC* overexpression in AML may be the increased activity of the transcription factor BRG1 (SMARCA4), which binds to chromatin and is part of the SWI/SNF protein complex [44,45]. It is known that the transcription factor BRG1 is essential for the survival and maintenance of the self-renewal potential of AML cells (mouse cell lines carrying the *cbfβ-myh11* or *mll-af9* mutation). Another reason for *C-MYC* overexpression in AML may be the increased expression of the *AML1* (*RUNX1*) gene. At least, a simultaneous increase in the expression levels of both genes is known to correlate with the pathogenesis of AML [46]. However, we did not determine the expression of *BRG1* and *AML1* genes.

It is interesting to note that this study revealed a more than 3-fold increase in the average level of *N-MYC* gene expression in the *FLT3-ITD-*positive group compared to the *FLT3-ITD*-negative group (*p* = 0.03946). At the same time, increased *N-MYC* oncogene expression was detected in the majority of patients (11 of 14, 78.5%) carrying the *FLT3-ITD* mutation. Previously, elevated *N-MYC* oncogene expression detected in children with AML and T-ALL has been shown to correlate with poor prognosis [19,47]. Only recently, it became known that, in mice with artificially induced myeloproliferative disease after injection of hematopoietic myeloid stem progenitors carrying the *flt3-itd* mutation, an increase in *N-MYC* oncogene expression was found [48]. *N-MYC* oncogene is known to promote stem cell self-renewal in BM and is one of the key genes whose transfection in healthy mice leads to AML development [19]. Recently, *N-MYC* overexpression was also found in children with neuroblastoma [49]. Later it was shown that overexpression of N-MYC oncogene in neuroblastoma cells is maintained with the participation of SPT16 transcription factor [50]. Both SPT16 and SSRP1 are part of the FACT (facilitates chromatin transcription) protein complex and participate in chromatin remodeling [51,52]. AURKA/B protein can also increase N-MYC expression in neuroblastoma. In this case, AURKA/B binds to N-MYC and prevents its ubiquitination with the participation of FBW7 ligase, which leads to the stabilization and accumulation of N-MYC in the cancer cell [53].

Interestingly, in the present study, we found a simultaneous 1.5-, 1.6-, 1.7-, 2.0-, and 2.6-fold increase in *SPT16*, *AURKA*, *C-MYC*, *N-MYC*, and *AURKB* gene expression in primary AML patients carrying a high (>0.5) *FLT3-ITD* mutation allelic load compared with low (<0.5) *FLT3-ITD* allelic load carriers. In contrast to all genes studied, whose expression correlated with increasing allelic load, *N-MYC* oncogene expression was moderately associated with low (<0.5: 0.15–0.49) allelic load and was independent of high (>0.5: 0.64–6.3) allelic load. It is known that *FLT3-ITD* mutation carriers with an allelic load of more than 0.5 have a markedly worse prognosis than patients with an allelic load of less than 0.5 [38]. Also, there is a view that the allelic load threshold (≥0.78) of the *FLT3-ITD* mutation is most suitable for an unfavorable prognosis. [7]. Some researchers believe that patients with a low (<0.5) *FLT3-ITD* allele load have a favorable prognosis if they are also carriers of *NPM1* gene mutations [38]. Other researchers refute this statement [54]. It is possible that the number of copies of the mutant *NPM1* gene could be relevant to the prognosis of *FLT3-ITD*-positive patients with different *FLT3-ITD* allelic load thresholds. However, in our case, this assumption may be true only in part. Thus, a reduced number (2–3, no multiplication by 100) of *NPM1/A* gene copies was found in 4 of 6 patients with a low (<0.5) allelic load of the *FLT3-ITD* mutation, whereas only 3 of 8 patients with a high (>0.5) allelic load of *FLT3-ITD* had an elevated number (11–25) of *NPM1/A* gene copies (data not shown). Thus, it is still unclear which additional causes may contribute to a worse prognosis in patients carrying the *FLT3-ITD* mutation. One such adverse phenomenon may be a 2-fold increase in *N-MYC* oncogene expression in carriers of the *FLT3-ITD* mutation with a high allelic load compared to carriers of *FLT3-ITD* with a low allelic load.

Biomarker monitoring in some *FLT3-ITD* carriers showed that patients with high allelic load and *N-MYC* (*SPT16*) overexpression were characterized by more pronounced resistance to treatment compared to *FLT3-ITD* carriers with low allelic load (Figure 5a–d). Thus, two patients (C, D), carriers of the *FLT3-ITD* mutation with a high allelic load, showed increasing resistance to treatment, in one case after gilteritinib therapy and in the other after allo-HSCT followed by therapy (FLAG+gilteritinib). At the same time, *N-MYC* expression levels were markedly elevated in both patients during the first 3 months after initiation of therapy, despite the reduced *FLT3-ITD* allelic load, which supports tumorigenesis. By analogy with neuroblastoma, we can assume that an additional transcription factor is required to maintain *N-MYC* oncogene expression in AML patients carrying the *FLT3-ITD* mutation. According to the results obtained on a limited group of patients with the *FLT3-ITD* mutation receiving therapy, we cannot claim that this factor is *SPT16*. However, it is interesting to note that both targets, *N-MYC* and *SPT16*, are involved in cohesin accumulation on chromatin, including the AT-rich pericentromeric region (where the *FLT3* gene on chromosome 13q12 is localized), which could lead to replication stress and promote carcinogenesis. Testing this hypothesis regarding the involvement of *N-MYC* (*SPT16*) in the pathogenesis of AML, resulting in cohesin accumulation and impaired DNA replication, requires additional experiments using cell lines derived from *FLT3-ITD*-positive AML patients.

## 5. Conclusions

The present work shows that *N-MYC* overexpression is extremely weakly related to or independent of allelic load of the *FLT3-ITD* mutation in AML patients. In some *FLT3-ITD* carriers after gilteritinib treatment, a decrease in *FLT3-ITD* mutation allelic load is not always accompanied by a decrease in *N-MYC* expression, but instead may lead to its overexpression. This is associated with the emergence of treatment resistance and leukemia progression. Thus, simultaneous determination of *FLT3-ITD* mutation allelic load and *N-MYC* oncogene expression level is reasonable for both disease onset and MRD monitoring during the first three months after therapy. This will allow to more effectively predict the course of AML and prevent the development of relapses. However, the question of what contributes to increased *N-MYC* expression levels independently of *FLT3-ITD* remains open.

## Figures and Tables

**Table 1:** Two groups of AML patients, according to the detected target mutations (*n* = 44).

The Group of Patients and the Frequency of Mutations	
*FLT3-ITD*-Negative	*FLT3-ITD*-Positive
t(8;21)(q22;q22) *AML1-ETO* ^†^, *n ′ =* 13, 29.5%; inv16(p13;q21) *CBFβ-MYH11* ^‡^, *n* = 8, 18.2%; normal karyotype, 46XX, *n* = 4, 46XY, *n* = 5, 20.4%	*FLT3-ITD* *^≠^, *n* = 14, 31.8%;

′—number of patients in the group, ^†^—*NPM1/A*, *n* = 3, ^‡^—*NPM1/A*, *n* = 2, *—*NPM1/A*, *n* = 7, ^≠^—1 of 14 patients had both *FLT3-ITD* and *FLT3-TKD* mutations.

**Table 2:** Achievement of remission and survival in *FLT3-ITD*-positive group, depending on insertion length and allelic load value.

Number of Patients in the Group, *n*		*FLT3-ITD* Mutation	χ^2^ Test, Fisher’s Test, *p*-Value
**Achievement of complete hematological remission**			
Yes	No	Insertion length	χ^2^, F, *p*
3	3	<39 bp	0.000, 1.0000, *p* > 0.05
4	4	>39bp	
**Survival**			
Alive	Dead	Insertion length	χ^2^, F, *p*
2	4	<39 bp	0.389, 0.67204, *p* > 0.05
4	4	>39 bp	
**Achievement of complete hematological remission**			
Yes	No	Allelic load	χ^2^, F, *p*
6	0	<0.5	10.500, 0.00233, *p* < 0.05
1	7	>0.5	
**Survival**			
Alive	Dead	Allelic load	χ^2^, F, *p*
5	1 ^†^	<0.5	7.024, 0.02564, *p* < 0.05
1	7	>0.5	

†—patient mortality was caused by SARS-CoV-2.

**Table 3:** Relative expression of the tested genes in *FLT3-ITD*-negative and *FLT3-ITD*-positive groups of AML patients.

Group of Patients	Number of Patients, *n*	Average Level of Gene Expression (N */*GAPDH*)	ANOVA with Bonferroni’s Post-Test: F-Stat, *p*-Value
***WT1***			
*FLT3-ITD*-negative	30	1442	8.1005, 0.008042
*FLT3-ITD*-positive	14	1625	
***C-MYC***			
*FLT3-ITD*-negative	30	380,538	9.2243, 0.005011
*FLT3-ITD*-positive	14	465,038	
***N-MYC***			
*FLT3-ITD*-negative	30	80,509	4.651, 0.03946
*FLT3-ITD*-positive	14	347,762	
***SPT16***			
*FLT3-ITD*-negative	30	77,468	4.6288, 0.0399
*FLT3-ITD*-positive	14	107,399	
***AURKA***			
*FLT3-ITD*-negative	30	286	1.8963, 0.179
*FLT3-ITD*-positive	14	425	
***AURKB***			
*FLT3-ITD*-negative	30	132	0.04112, 0.8407
*FLT3-ITD*-positive	14	316	

*—number of copies of the gene under study.

## Data Availability

The datasets presented in this article are not publicly available as all patient data are anonymized.

## Supplementary Materials

The following supporting information can be downloaded at: https://www.mdpi.com/article/10.3390/pathophysiology30030024/s1. Table S1—Information on the comorbidities and sex of the study patients. Table S2—Nucleotide sequences of commercially synthesized oligonucleotides selected using the NCBI system. Table S3—Evaluation of relative expression levels of the studied genes (*WT1, C-MYC, N-MYC, SPT16, AURKA, AURKB*) and *FLT3-ITD* mutation including *FLT3-ITD* allele load in primary AML patients. Table S4—Biomarker monitoring in *FLT3-ITD* mutation carriers (n = 4) during therapy.
